# Triptolide attenuates proteinuria and podocyte apoptosis via inhibition of NF-κB/*GADD45B*

**DOI:** 10.1038/s41598-018-29203-1

**Published:** 2018-07-18

**Authors:** Ling Wang, Liwen Zhang, Qing Hou, Xiaodong Zhu, Zhaohong Chen, Zhihong Liu

**Affiliations:** National Clinical Research Center of Kidney Diseases, Jinling Hospital, Nanjing University School of Medicine, Nanjing, 210016 China

## Abstract

Podocyte injury is a primary contributor to proteinuria. Triptolide is a major active component of Tripterygium wilfordii Hook F that exhibits potent antiproteinuric effects. We used our previously developed *in vivo* zebrafish model of inducible podocyte-target injury and found that triptolide treatment effectively alleviated oedema, proteinuria and foot process effacement. Triptolide also inhibited podocyte apoptosis in our zebrafish model and *in vitro*. We also examined the mechanism of triptolide protection of podocyte. Whole-genome expression profiles of cultured podocytes demonstrated that triptolide treatment downregulated apoptosis pathway-related *GADD45B* expression. Specific overexpression of *gadd45b* in zebrafish podocytes abolished the protective effects of triptolide. *GADD45B* is a mediator of podocyte apoptosis that contains typical NF-κB binding sites in the promoter region, and NF-κB p65 primarily transactivates this gene. Triptolide inhibited NF-κB signalling activation and binding of NF-κB to the *GADD45B* promoter. Taken together, our findings demonstrated that triptolide attenuated proteinuria and podocyte apoptosis via inhibition of NF-κB/*GADD45B* signalling, which provides a new understanding of the antiproteinuric effects of triptolide in glomerular diseases.

## Introduction

Podocytes are specialized epithelial cells of the glomerulus that crucially contribute to the filtration apparatus of the kidney. Podocyte damage inevitably leads to proteinuria and glomerular dysfunction, which are relevant to acute and chronic glomerular diseases, including focal segmental glomerulosclerosis (FSGS), diabetic kidney disease (DKD) and HIV-related nephropathy^1,2^. However, treatments targeting podocytes are still very limited.

The *GADD45* gene family is involved in the regulation of cell survival and apoptosis, DNA damage repair, and cell cycle arrest. Shi *et al*. found that *GADD45B* was upregulated in glomeruli of podo-Dicer^−/−^ mice, which indicated the involvement of *GADD45B* in podocyte damage^3^. We previously found that *GADD45B* was a critical mediator of podocyte apoptosis and that this gene was significantly upregulated in podocytes of patients with FSGS. Podocyte-specific overexpression of *gadd45b* in zebrafish podocytes aggravated proteinuria and foot process effacement as well as promoted podocyte apoptosis via activation of the p38 MAPK pathway^4^. These results suggest that *GADD45B* is a potent therapeutic target for the management of podocyte injury.

Triptolide is a small biologically active molecule and key component isolated from the Chinese medicinal herb Tripterygium wilfordii Hook F. Triptolide exhibits multiple pharmacological benefits, including anti-inflammatory, immunosuppressive, and antineoplastic effects^5,6^. Triptolide obviously decreases proteinuria levels in patients with minimal change disease (MCD), focal segmental glomerulosclerosis (FSGS) and membranous nephropathy (MN)^7^. Triptolide reduces proteinuria in rats with puromycin-induced nephropathy (PAN) and passive Heymann nephritis via inhibition of ROS production and the p38 MAPK signalling pathway as well as restoration of RhoA activity^8,9^. However, the key targets of triptolide in podocytes are not known.

Zebrafish (*Danio rerio*) emerged as an ideal *in vivo* model for podocyte research^10,11^ and small molecule screening^12,13^. We used an inducible podocyte-target injury model in transgenic zebrafish as an *in vivo* model system and cultured human podocytes as an *in vitro* model to examine the protective mechanism of triptolide in podocyte injury. This transgenic zebrafish model is a podocyte-specific ablation model that exhibits no toxic effects on any other cells compared to the PAN nephrosis rat model. Our results indicated that triptolide attenuated proteinuria and podocyte apoptosis in zebrafish. These effects were associated with inhibition of *GADD45B* expression. We also found that triptolide inhibited NF-κB p65 phosphorylation, which binds to the *GADD45B* promoter and activates *GADD45B* transcription *in vitro*.

## Results

### Triptolide attenuates MTZ-induced oedema and proteinuria in zebrafish

**Figure 1:**
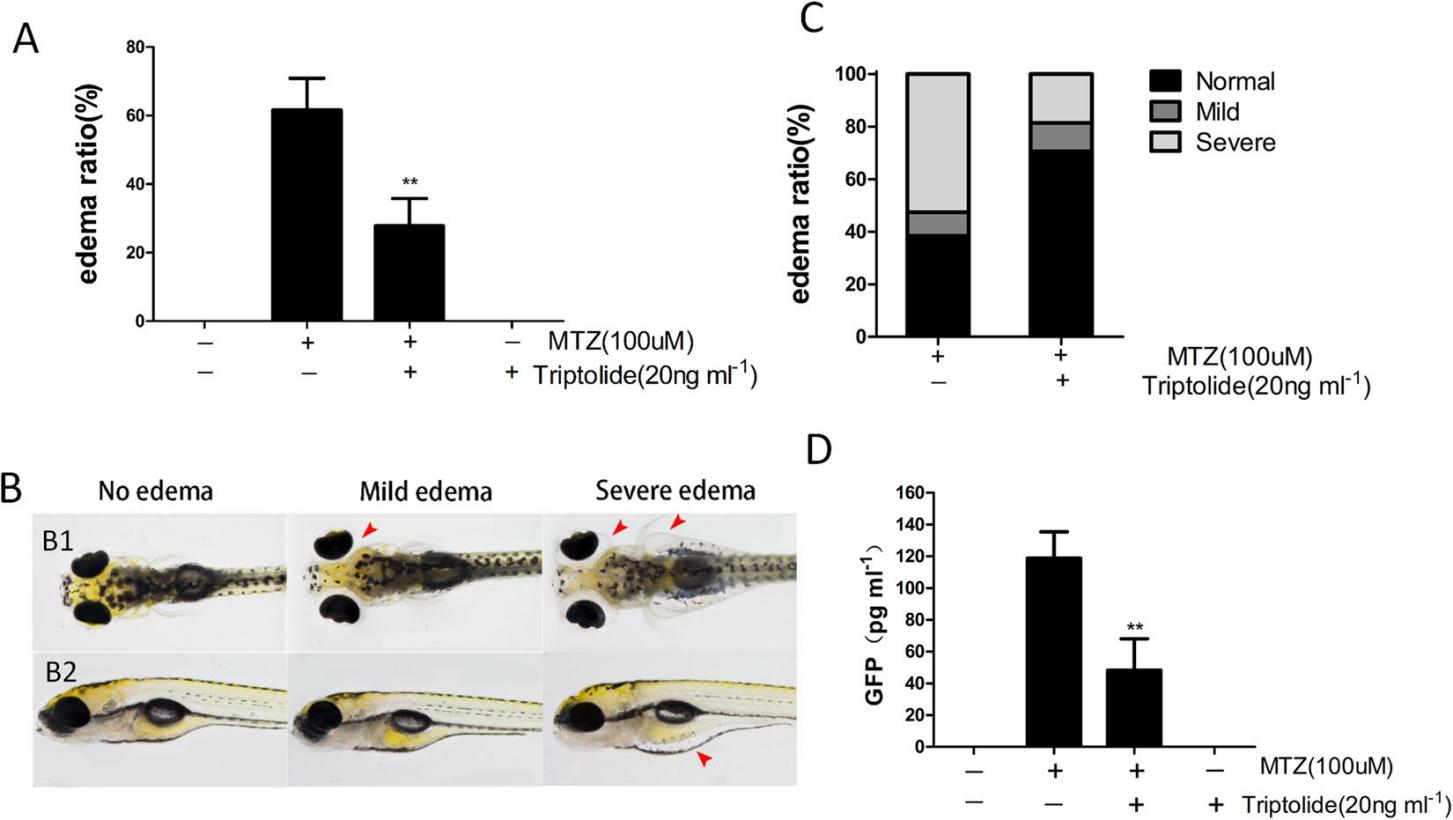
Triptolide treatment alleviated MTZ-induced oedema and proteinuria. (**A**,**C**) Quantification of the oedema ratio (n = 30) and severity of oedema. The embryos were treated with 100 μM MTZ from 84 hpf to 132 hpf. Triptolide pretreatment obviously reduced MTZ-induced oedema percentage and severity. (**B**) Representative figure shows the severity of oedema (arrowhead) from a dorsal view (B1) and side view (B2). (**D**) GFP Quantification using ELISA showing that triptolide pretreatment reduced the MTZ-induced proteinuria (n = 3). **P < 0.01. (On the basis of three triplicate tests).

We initially evaluated the effects of triptolide at 0, 20, 40, 60, 80, 100, 120 and 200 ng ml^−1^ via treatment of wild-type zebrafish embryos at 60 hpf for 72 h. We found that the embryos exhibited several development abnormalities, including body curve, growth delay and axis shortening at triptolide concentrations greater than 80 ng ml^−1^ (see Supplement Fig. 1). None of the embryos survived in 200 ng ml^−1^ triptolide. Triptolide at 20, 40 and 60 ng ml^−1^ produced no obvious toxic effects on embryos. The bacterial nitroreductase (NTR) in the *Tg*(*pod:gal4;UAS:NTR-mCherry*) is specifically expressed in podocytes of zebrafish pronephros and mesonephros, which convert metronidazole (MTZ) into a cytotoxin to induce only podocytes injury^14^. The *Tg*(*pod:Gal4;UAS:NTR-mCherry*) embryos (84 hpf) were exposed to 100 μM MTZ for 48 h to induce podocyte injury. The transgenic larvae exhibited periorbital oedema (POE) and sac yolk oedema (SE) (Fig. 1B). MTZ treatment induced a total oedema ratio of 61.5 ± 18.4%. We categorized the embryos with POE alone as mild oedema and embryos with POE and SE as severe oedema (Fig. 1B). The mild and severe oedema ratios were 8.9 ± 4.1% and 52.6 ± 25.3%, respectively. We pre-treated the embryos (60 hpf) with 20 ng ml^−1^ triptolide for 24 h prior to MTZ treatment to investigate the effect of triptolide on transgenic zebrafish carrying NTR-mCherry. The total oedema ratio decreased significantly to 27.8% ± 15.9% compared to the MTZ group (Fig. 1A). The mild and severe oedema ratios were 10.1 ± 5.9% and 18.5 ± 10.8%, respectively (Fig. 1C). We performed a proteinuria assay in *Tg*((*pod:Gal4;UAS:NTR-mCherry;lfabp:VDBP-GFP*) embryos (120 hpf)^10^ using ELISA against GFP after MTZ treatment to examine whether triptolide attenuated proteinuria. Cotreatment with triptolide significantly reduced MTZ-induced proteinuria (Fig. 1D). Therefore, triptolide treatment significantly reduced the percentage of larvae that developed oedema and proteinuria.

### Triptolide protects podocytes from MTZ-induced injury in zebrafish

**Figure 2:**
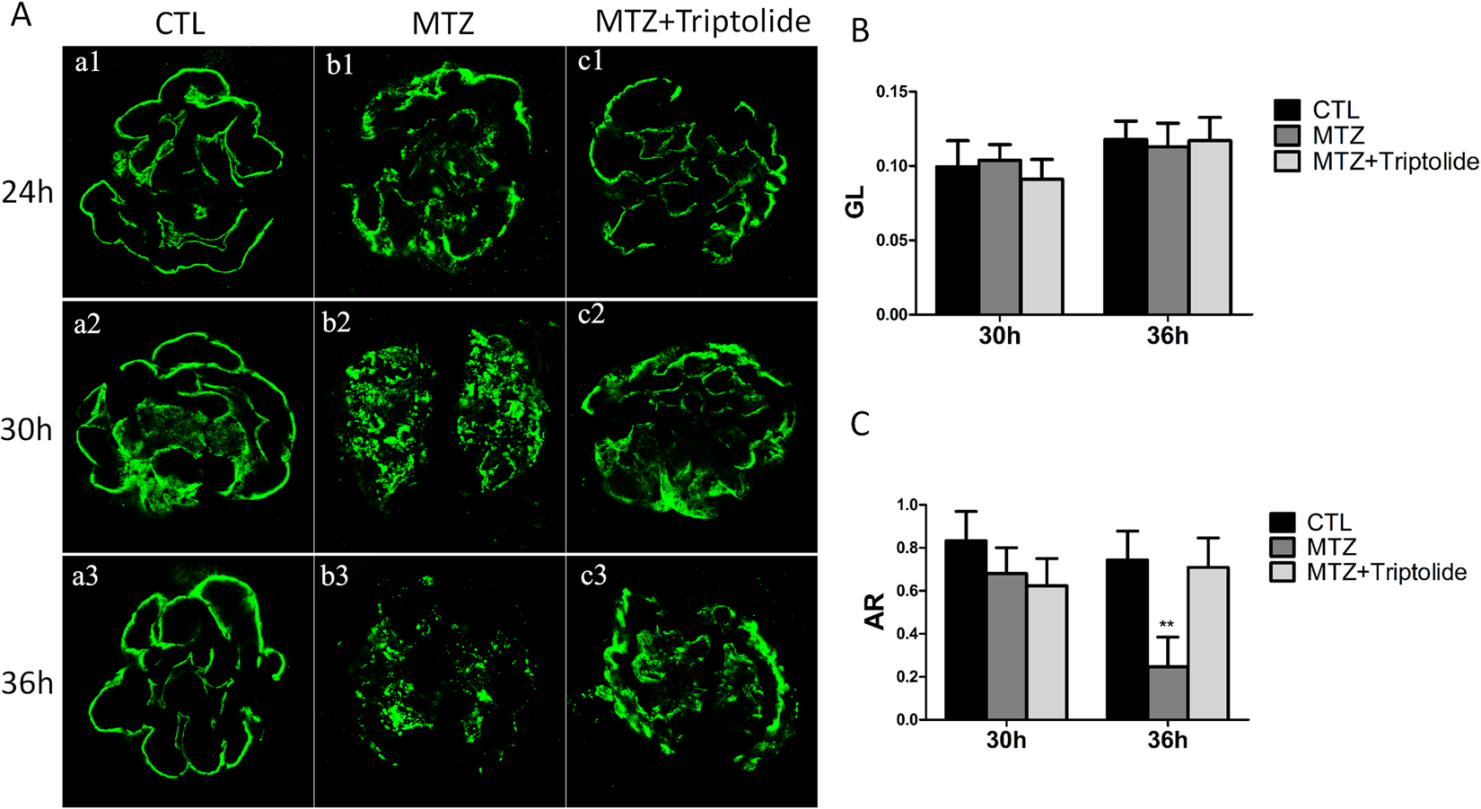
The effects of triptolide on podocin expression and distribution in MTZ-treated Tg(*pod:gal4;UAS:NTR-mCherry*) zebrafish. (**A**) The expression and distribution of podocin in different groups was measured and examined at 24 h, 30 h and 36 h after MTZ treatment in 84 hpf larvae; (a1–a3) normal control (CTL); (b1–b3) MTZ model group; (c1–c3) MTZ + triptolide group. (**B**) No obvious mean immunofluorescence intensity (GL) differences were observed between the three groups at 30 h and 36 h. (**C**) Positively stained area to the glomerular area (AR) revealed a remarkably reduction at 36 h in the MTZ model group, but remained strong in the triptolide treatment group. (n = 3), **P < 0.01, (original magnification, x1000).

Podocin is a protein component of the filtration slits of podocytes, and it exhibits a very fine linear-like pattern along the capillary loop in normal zebrafish pronephros glomeruli (Fig. 2A(a1–a3). Mutations in *NPHS*2 or the loss of podocin produce nephritic syndrome and proteinuria^15^. We created a polyclonal anti-zebrafish podocin antibody and performed whole-mount antibody staining to investigate the effect of triptolide on podocin expression in zebrafish. We divided embryos into an MTZ group and triptolide plus MTZ group. Embryos treated with MTZ for 24 h exhibited a discontinuous distribution of podocin, and the distribution of podocin remained intact in the triptolide + MTZ group (Fig. 2A(b1,c1). Podocin expression was detached in an obvious coarse granular pattern in the MTZ group after 30 h of treatment, but exhibited a linear-like distribution in the triptolide + MTZ group (Fig. 2A(b2,c2). A serious decline in podocin expression was observed in the MTZ group compared to the triptolide + MTZ group after 36 h of MTZ treatment (Fig. 2A(b3,c3). Quantification demonstrated that the immunofluorescence intensity (GL) and stained area to the glomerulus area (AR) was not altered significantly in either group at 30 h (Fig. 2B,C). Podocin AR decreased remarkably at 36 h in the MTZ group (P < 0.01) but did not noticeably change in the triptolide + MTZ group (Fig. 2B,C).

**Figure 3:**
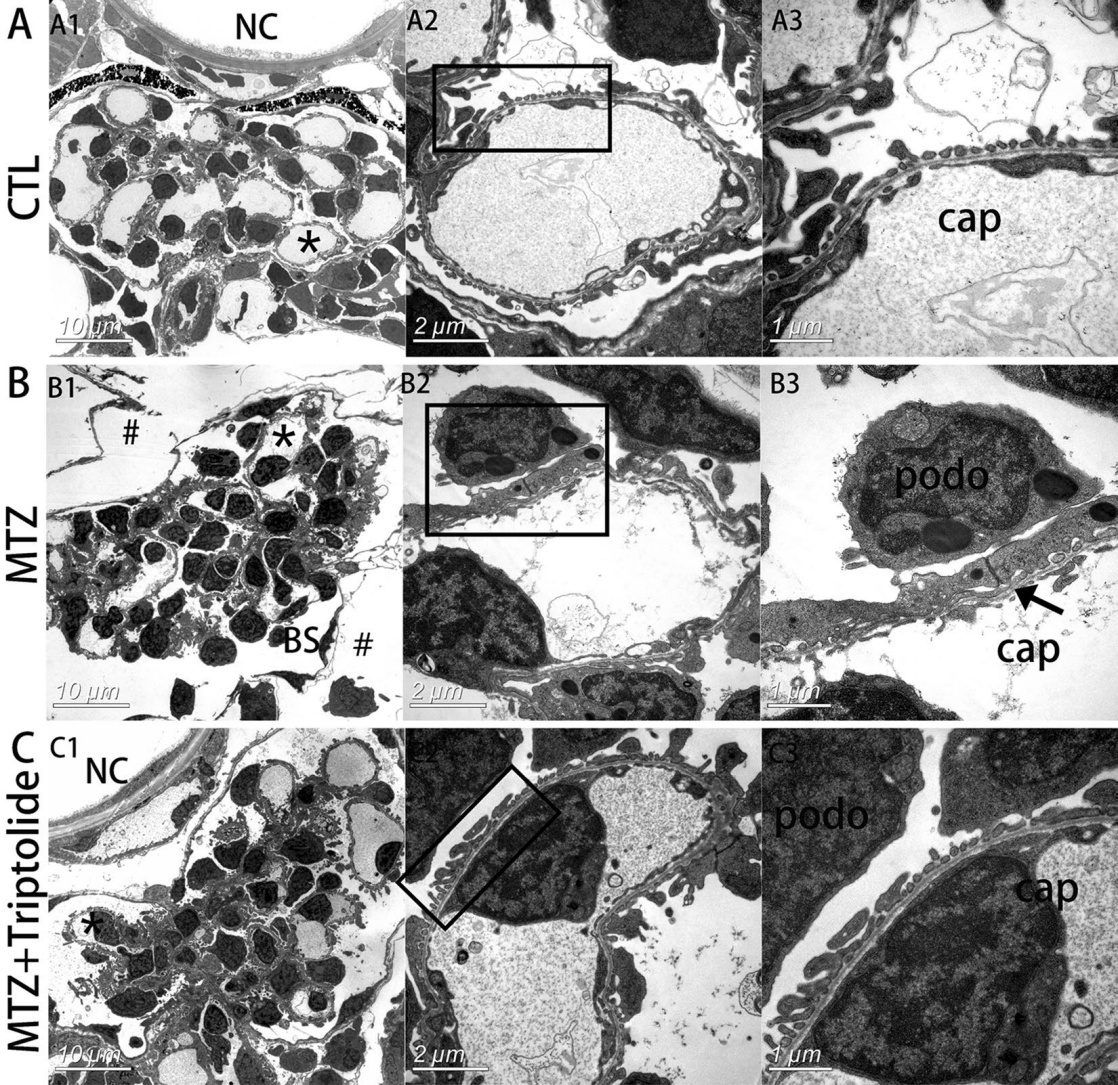
Triptolide reversed podocyte foot process effacement in MTZ-treated Tg(*pod:gal4;UAS:NTR-mCherry*) zebrafish. (**A**) Electron micrograph of normal glomerular vascular loop morphology and foot process development at 6 dpf in wild-type larvae. (**B**) Glomerular morphology of zebrafish treated with MTZ for 24 h. At low magnifications, an enlarged space outside of Bowman’s capsule was observed, indicating oedema (B1, indicated by #). Extensive foot process effacement was observed at higher magnifications (B3, indicated by arrows). (**C**) Triptolide prevented most foot process effacement. (n = 3). *Capillary that was magnified. podo, podocyte; cap, capillary space; BS, Bowman’s space; NC, notochord. Scale bar = 10 μm (A1,B1,C1), 2 μm (A2,B2,C2), 1 μm (A3,B3,C3).

Transmission electron microscopy (TEM) revealed that the space outside of Bowman’s capsule was enlarged in the MTZ group (Fig. 3B1,#), which indicates oedema. Higher magnifications revealed the disappearance of slit diaphragms and severe foot process effacement in the MTZ group (Fig. 3B2,B3). However, the space around glomeruli was normal, and foot process effacement was intact in the triptolide + MTZ group (Fig. 3C). Therefore, we concluded that triptolide pre-treatment protected podocytes from MTZ-induced injury and prevented podocyte loss.

### Triptolide protects podocytes from apoptosis in MTZ-induced zebrafish and PAN- treated podocytes

**Figure 4:**
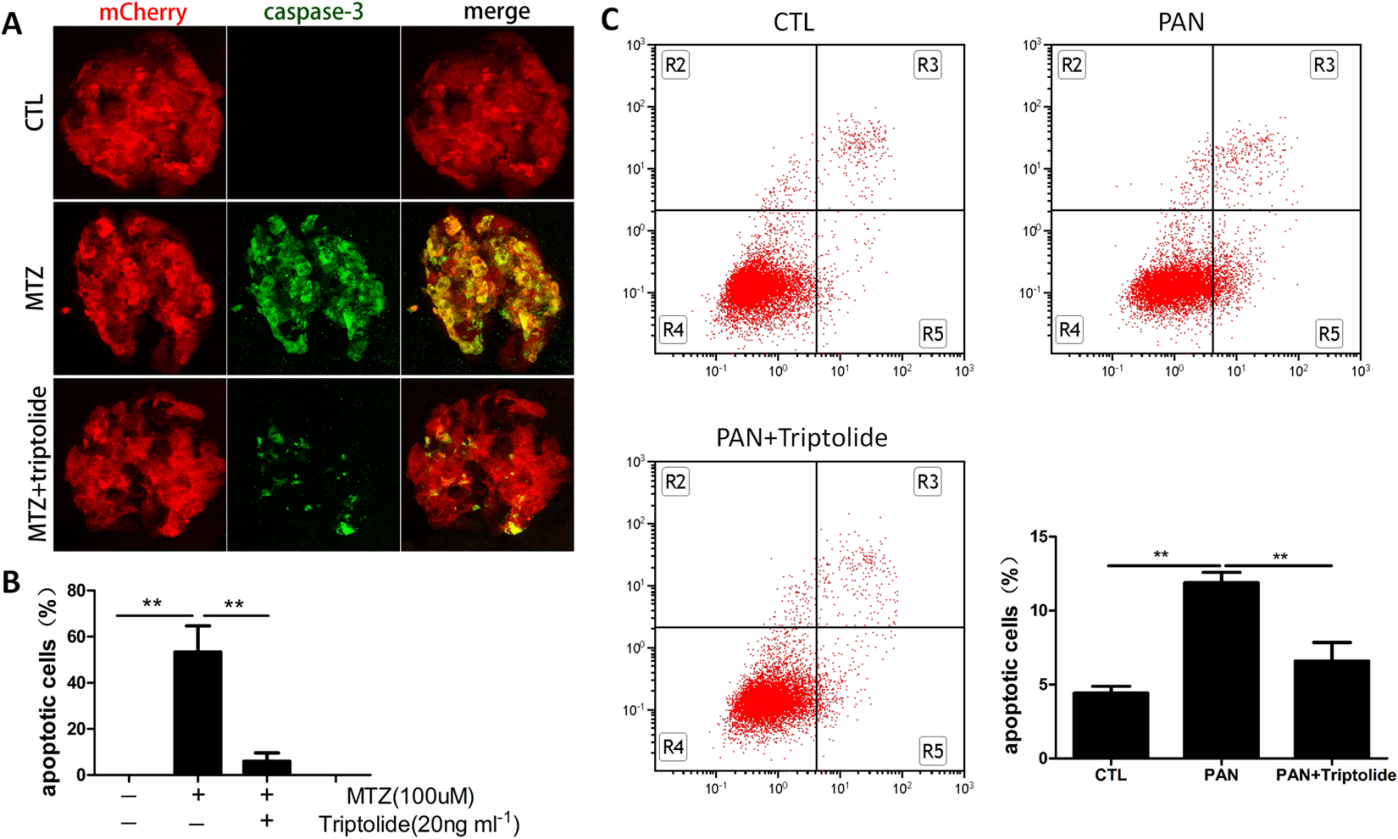
Triptolide suppressed podocyte apoptosis. (**A**) 84 hpf larvae treated with MTZ for 24 h exhibit remarkable podocyte apoptosis signalling compared to the untreated group (CTL) on immunostains against cleaved caspase-3. The number of apoptotic cells was significantly reduced in the triptolide treatment group. (**B**) Quantification of caspase-3-stained area (green) to mCherry (red). Data are expressed as the means ± SEM. The confocal images are shown at a maximum intensity projection. (n = 3), **P < 0.01; *P < 0.05. (Original magnification, x1000). (**C**) Annexin V-FITC/PI staining followed by FCM showed podocytes apoptosis induced by PAN was alleviated by triptolide. **P < 0.01. (On the basis of three triplicate tests).

Apoptosis is the process of programmed cell death that occurs in multicellular organisms. Previous studies demonstrated that apoptosis increased in MTZ-treated zebrafish podocytes^10^. We performed activated caspase-3 staining to examine the protective function of triptolide on podocyte apoptosis. We confirmed extensive apoptosis in podocytes following 24 h of MTZ treatment, and capase-3 signals co-localized with mCherry in pronephros. No activated caspase-3 was observed in the normal group. However, we pre-treated Tg(*pod:Gal4;UAS:NTR-mCherry)* embryos with triptolide for 24 h prior to MTZ treatment, and few apoptosis signals were detected in zebrafish pronephros (Fig. 4A). We quantified the number of apoptotic podocytes and measured the area ratio of capase-3 to mCherry, which represented all podocytes. The percentage of apoptotic podocytes were 53.2 ± 19.8% in the MTZ group and 5.9 ± 3.6% in triptolide + MTZ group (Fig. 4B). Moreover, *in vitro* study showed the increase in podocytes apoptosis challenged with PAN was mitigated with triptolide treatment (Fig. 4C). These results suggest the protective effect of triptolide on podocytes directly.

### Genome-wide transcriptome analysis in triptolide-treated human podocytes

**Figure 5:**
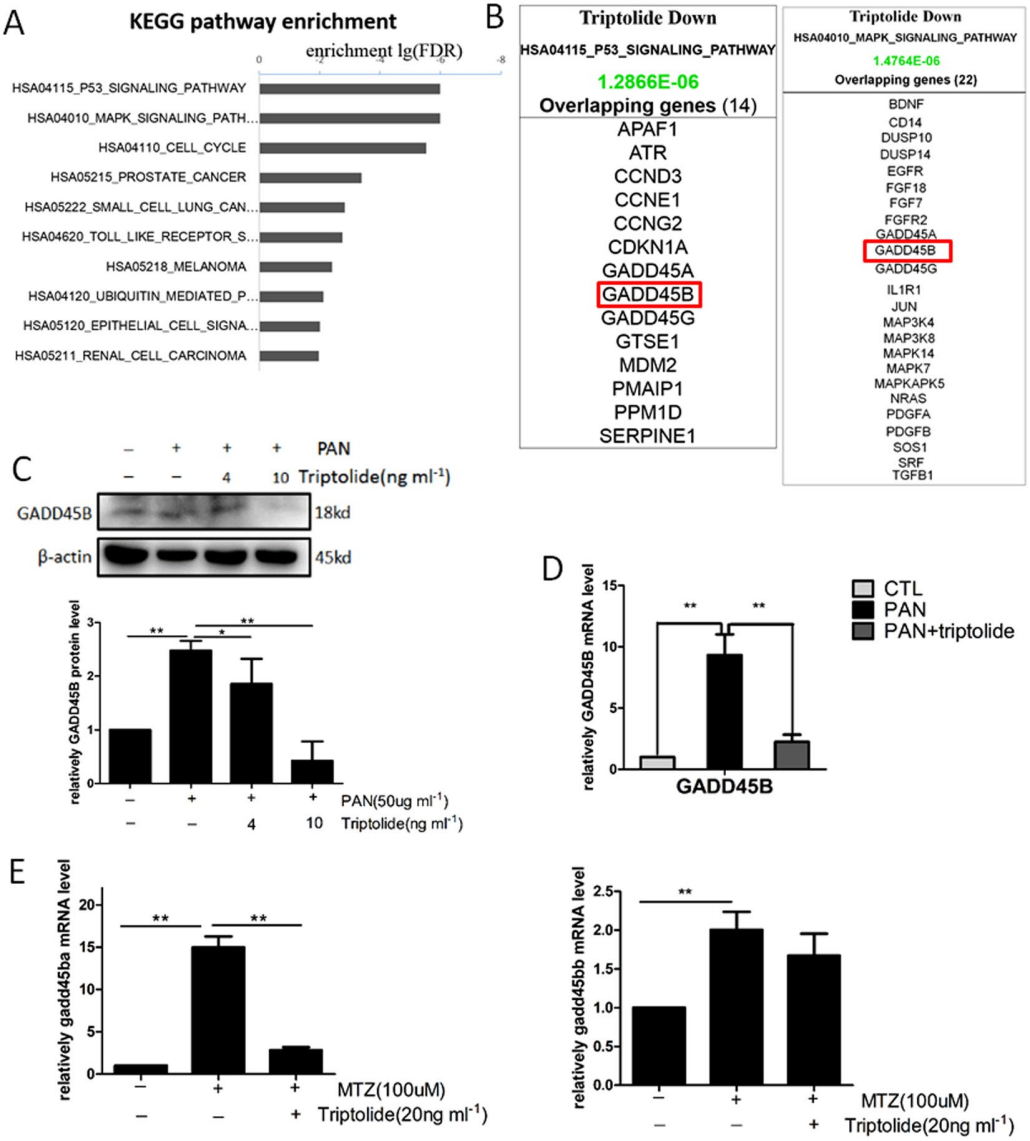
Triptolide downregulates podocyte *GADD45B* expression *in vitro* and *in vivo*. (**A**) KEGG pathway enrichment analysis of triptolide-treated human podocytes. The most significant pathways regulated by triptolide were the *p53* and MAPK signalling pathways. (**B**) Triptolide downregulated *GADD45B* expression, which is involved in *p53* and MAPK signalling. (**C**,**D**) Western blots and qRT-PCR demonstrated that GADD45B expression was upregulated after PAN (50 µg ml^−1^) treatment for 12 h and inhibited by pretreatment with 10 ng ml^−1^ triptolide. (**E**) Measurement of GADD45B expression in isolated zebrafish glomeruli following MTZ induction of podocyte injury. Triptolide significantly decreased *gadd45ba* expression but had no effect on *gadd45bb*. Data are expressed as the means ± SEM. (n = 3), **P < 0.01; *P < 0.05. (On the basis of three triplicate tests). Each blot was cropped at the position of the blotted protein, and high-contrast was not used.

We performed a genome-wide transcriptome microarray on RNA samples extracted from triptolide-treated and non-treated human podocytes to further elucidate the intracellular signalling pathways for the effects of triptolide. We performed KEGG Pathway Analysis to delineate the sensitivity of certain pathways or biological processes to triptolide. The results revealed that the most affected signalling pathways after triptolide treatment to podocytes were the *p53* and MAPK signalling pathways (Fig. 5A). Both of these pathways mediate cell apoptosis, which further indicated that triptolide regulated podocyte apoptosis. The downregulation of *GADD45B* perfectly overlapped with these two signalling pathways (Fig. 5B).

### Triptolide suppresses podocyte *GADD45B* expression *in vivo* and *in vitro*

Recent studies demonstrated that specific over-expression of *gadd45ba/b* in zebrafish podocytes increased MTZ-induced apoptosis, and knockdown of *gadd45ba/b* inhibited apoptosis^4^. We examined podocyte *GADD45B* expression at the protein and mRNA level *in vitro* and *in vivo* to validate the results of the genome-wide transcriptome microarray. PAN induced podocyte injury *in vitro*, and GADD45B mRNA and protein expression in the PAN-treated group increased significantly compared to the untreated group. Triptolide remarkably decreased GADD45B expression at 10 ng ml^−1^ in PAN-cultured podocytes (Fig. 5C and D). We treated *Tg(pod:Gal4;UAS:NTR-mCherry)* embryos with MTZ and MTZ + triptolide and isolated glomeruli from both groups. Triptolide remarkably downregulated *gadd45ba* expression compared to the MTZ group, but there was no effect on *gadd45bb* (Fig. 5E).

### Overexpression of *gadd45b*a in zebrafish podocytes abolishes the protective effects of triptolide

**Figure 6:**
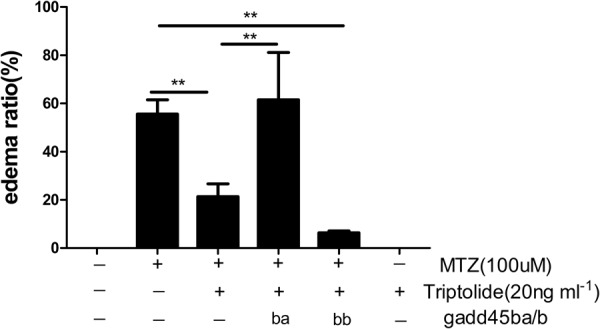
Overexpression of gadd45ba in zebrafish podocyte abolished the protective effects of triptolide. 84 hpf Tg(*pod:Gal4;UAS:NTR-mCherry;UAS:gadd45ba/b*,*cmlc2:GFP*) larvae, which overexpress gadd45ba/b specifically in podocytes, were exposed to MTZ (100 µM) following pretreatment with triptolide (20 ng ml^−1^) for 48 h. The oedema ratio revealed that triptolide significantly decreased the total oedema ratio, but this effect was abolished by overexpression gadd45ba in podocytes. gadd45bb overexpression did not exhibit this function. (n = 3). **P < 0.01.

We previously generated a transgenic zebrafish model of podocyte-specific *gadd45b*a/b overexpression^4^. We treated this triple transgenic zebrafish Tg(*pod:Gal4;UAS:NTR-mCherry;UAS:gadd45ba*/*b*,*cmlc2:GFP*) with MTZ after a 24 h triptolide pretreatment to further investigate whether triptolide protected podocytes from injury via inhibiting *GADD45B* expression. We counted oedema ratios after a 48 h exposure. The percentage of oedema was not decreased in podocytes overexpressing *gadd45ba* compared with the effect of triptolide in the Tg(*pod:Gal4;UAS:NTR-mCherry*) group. However, *gadd45bb* overexpression did not produce this result (Fig. 6). These results clearly indicate that triptolide regulation of *GADD45B* is a critical mechanism that confers the anti-proteinuria function of triptolide.

### Triptolide inhibits *GADD45B* transcriptional activation via regulation of NF-κB

**Figure 7:**
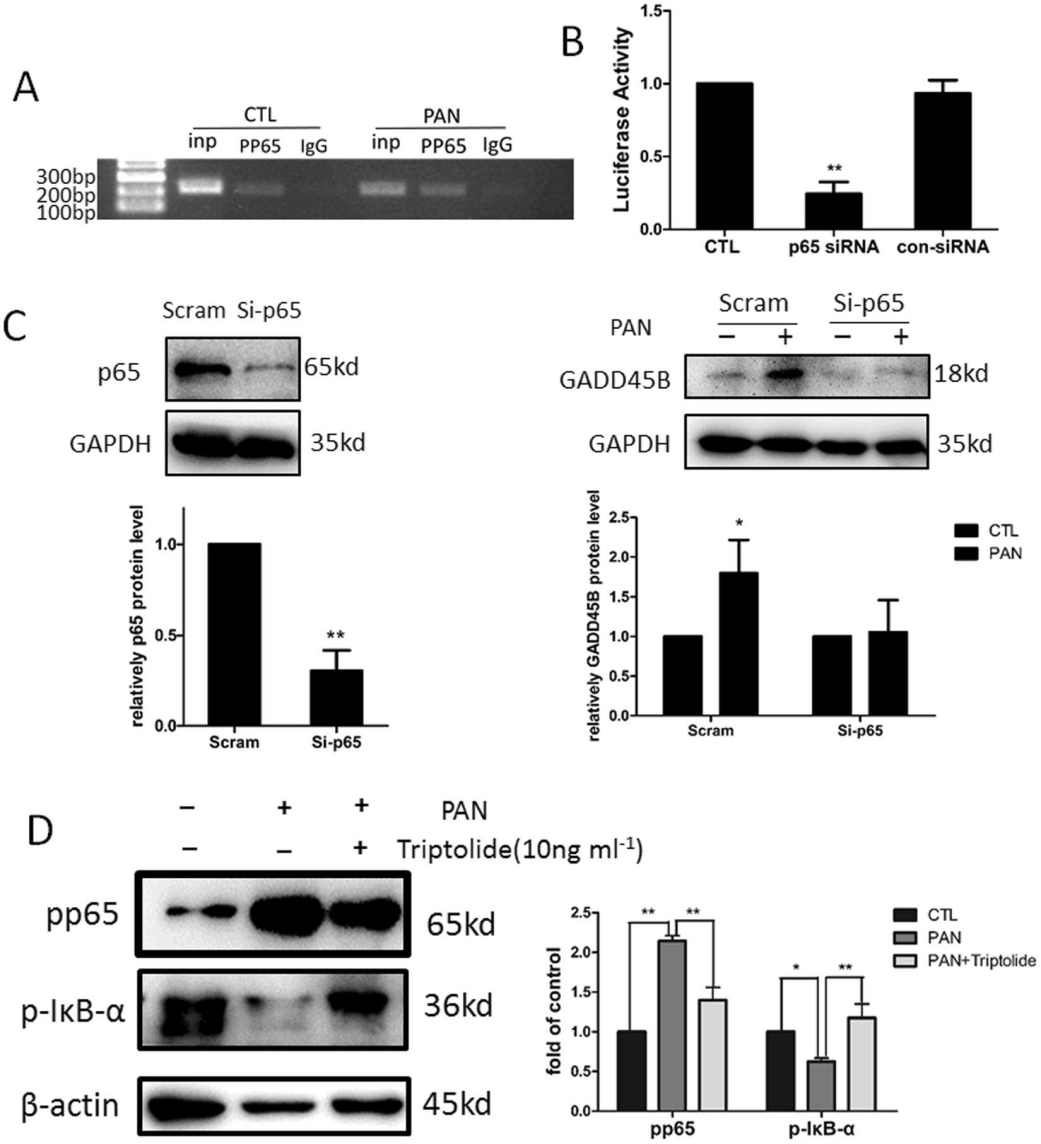
Triptolide downregulates *GADD45B* expression via inhibition of NF-κB signalling. (**A**) Chromatin immunoprecipitation (ChIP) analysis in podocytes using an antibody to phospho-NF-κB p65, followed by polymerase chain reaction (PCR) using the *GADD45B* gene promoter-specific primer. The amplified sequence is designed +255 to +455 base pairs downstream of the transcription start site (chr19: 2476721-2476731). More NF-κB p65 bound directly to the *GADD45B* promoter region after exposure to PAN for 24 h compared to normal control. (**B**) The luciferase assay confirmed that *GADD45B* promoter activity was significantly decreased by inhibition of NF-κB p65. (**C**) Human podocytes transfected with NF-κB p65 siRNA and scramble control for 48 h followed by PAN stimulation (50 µg ml^−1^) for 12 h. NF-κB p65 knockdown prevented PAN-induced upregulation of GADD45B. (**D**) Human podocytes were incubated with 50 µg ml^−1^ PAN and 10 ng ml^−1^ triptolide for 6 h, and immunoblotting shows a marked reduction in NF-κB activity in the podocytes treated with triptolide and PAN compared to podocytes treated with PAN only. (n = 3). **P < 0.01; *P < 0.05. Each blot was cropped at the position of the blotted protein, and high-contrast was not used.

In *silico* analysis identified several NF-κB consensus sequences within the *GADD45B* gene (http://www.sabiosciences.com/)). P65 (RelA) is the only TAD-domain-containing subunit that activates the transcriptional expression of *GADD45B* via binding to three κB elements on the promoter region of the *GADD45B* gene^16^. Triptolide is an effective inhibitor that is involved in the activation of NF-κB signalling^17–20^. Therefore, we determined whether triptolide regulated *GADD45B* expression via suppression of NF-κB in podocytes. Chromatin immunoprecipitation analysis was performed to determine whether phosphorylated NF-κB p65 bound to the *GADD45B* promoter region in chromatin of podocytes with or without PAN treatment. The results demonstrated that phosphorylated NF-κB p65 directly bound to the +255 to +455 region downstream of the transcription start site. Phosphorylated NF-κB p65 antibodies immunoprecipitated more chromatin in cells incubated with PAN than untreated cells (Fig. 7A). A double luciferase reporter assay was also performed in podocytes. We constructed a human *GADD45B* promoter luciferase plasmid and transfected it into human podocytes cultured at 33 °C. Reporter luciferase activity was significantly decreased in cultured podocytes co-transfected with NF-κB p65 siRNA (Fig. 7B). We transfected podocytes with NF-κB p65 siRNA to knockdown its expression for 48 h prior to PAN treatment (50 µg ml^−1^) for 12 h to further investigate the effect of NF-κB inhibition on GADD45B expression. NF-κB p65 knockdown using siRNA effectively prevented PAN-induced upregulation of GADD45B (Fig. 7C). These results indicated that NF-κB p65 transactivated *GADD45B* expression in podocytes. We examined whether triptolide inhibited NF-κB signalling in podocytes using Western blotting. Our data demonstrated higher protein levels of phosphorylated NF-κB p65 and lower phosphorylated IκB-α levels in the PAN-treated group *in vitro* compared to the normal group. Triptolide treatment significantly reduced phosphorylated NF-κB p65 protein levels and enhanced phosphorylated IκB -αlevels (Fig. 7D). These results indicate that triptolide regulates *GADD45B* expression via inhibition of NF-κB signalling.

## Discussion

The present study demonstrated several novel results. First, triptolide attenuated proteinuria and protected podocytes from apoptosis in a zebrafish model. Second, this effect was associated with inhibition of *GADD45B* expression, which mediates podocyte apoptosis via activation of the p38 MAPK signalling pathway. Third, triptolide regulated *GADD45B* expression via suppression of NF-κB activation, which bound to the *GADD45B* promoter and activated its transcription.

The mechanism of triptolide protection of podocytes was investigated previously^8,9,21,22^. However, the definitive pharmacological target of triptolide in podocytes remained controversial. The present study provided a novel mechanism. We discovered that triptolide suppressed podocyte apoptosis. Many stimulating factors induce podocyte apoptosis, including inflammation and ROS production. Triptolide also exhibits strong anti-inflammatory activities^23^ and inhibits ROS generation^8,9^. Our previous research demonstrated that p38 MAPK signalling was the key regulatory pathway for triptolide efficacy. The present study elucidated the underlying mechanism of triptolide alleviation of podocyte apoptosis and the upstream signalling of the p38 MAPK pathway. We recently demonstrated that *GADD45B* exacerbated apoptosis in zebrafish podocytes and *in vitro*. *GADD45B* was confirmed as an upstream target protein of the p38 MAPK signalling pathway in the mediation of apoptosis. Triptolide and *GADD45B* exhibit many opposite properties, and microarray analysis indicated that triptolide downregulated *GADD45B* expression. Therefore, we validated the potential connection between triptolide podocyte protection and its regulation of *GADD45B* in an inducible zebrafish podocyte injury model. Our results confirmed that triptolide treatment inhibited *GADD45B* expression, and *gadd45ba* overexpression in zebrafish podocytes abolished the beneficial effect of triptolide. Therefore, we suggest that *GADD45B* is an essential molecule that, at least partially, mediates the triptolide-podocyte protective function.

The zebrafish *gadd45b* gene has two members, *gadd45b*a and *gadd45bb*. The present study demonstrated that triptolide inhibited *gadd45ba* expression but did not affect *gadd45bb* expression, and *gadd45bb* overexpression did not affect the protective function of triptolide. Our previous studies revealed that normal zebrafish glomeruli express more *gadd45ba* than *gadd45bb* and upregulation of *gadd45ba* was more prominent than *gadd45bb* after podocyte injury. Overexpression of *gadd45ba* in zebrafish podocytes resulted in a more serious phenotype than *gadd45bb* overexpression. These results suggest that *gadd45ba*, but not *gadd45bb*, plays a primary role in zebrafish podocytes. Therefore, triptolide protects zebrafish podocytes from injury primarily via inhibition of *gadd45ba* expression rather than *gadd45bb* expression.

The molecular events that lead to *GADD45B* upregulation after podocyte injury are still not fully understood. LPS^24^, TNFα^24^ and interleukin 1(IL-1)^25^ activate NF-κB signalling and induce *GADD45B* expression. The *GADD45B* promoter contains three NF-κB binding sites, and each of these sites is required for optimal transcriptional activation^16^. SiRNA knockdown of NF-κB p65 reduced GADD45B expression in PAN-induced podocyte injury. ChIP-PCR revealed that phospho-NF-κB p65 bound to the *GADD45B* promoter in human podocytes, and more phospho-NF-κB P65 bound to the *GADD45B* promoter after podocyte injury compared to the normal group. The luciferase assay further confirmed that co-transfection with NF-κB p65 siRNA strongly reduced *GADD45B* promoter activity. Therefore, *GADD45B* induction by various stimuli in podocytes was partially due to increased NF-κB activity, which is required for *GADD45B* transcriptional activation. Many previous studies demonstrated that triptolide was a potent inhibitor of NF-κB. We also found that triptolide inhibited NF-κB activation via increasing phospho-IκBα and reducing NF-κB p65 phosphorylation at the protein level following podocyte injury. These findings suggest that triptolide regulates *GADD45B* transactivation via inhibition of NF-κB signalling.

The present study used the transgenic zebrafish line Tg (*pod:Gal4;UAS:NTR-mCherry)* to establish an inducible podocyte injury model that exhibits cell death exclusively within NTR^+^ cells^14,26^. Triptolide also produced remarkably beneficial effects on zebrafish podocytes, which consistent with the results in a puromycin aminonucleoside nephrosis rat model. Our results provide novel evidence for the clinical application of triptolide. However, this zebrafish podocyte injury model is a podocyte-specific ablation model that exhibits no toxic effect on any other cells, compared to the PAN nephrosis rat model. Many methods are used to estimate glomerular structure and function. We monitored proteinuria in the transgenic zebrafish line Tg(*pod:Gal4;UAS:NTR-mCherry;lfabp:vbp-gfp*), which expresses a VDBP-GFP fusion protein of a similar molecular weight as mammalian albumin^10^. *In situ* hybridization, RT-PCR, and the synthesized antibody examined alterations in nephrin and podocin at the protein level^27^. We also performed podocin staining to observe its expression and distribution after triptolide treatment. TEM allowed us to observe morphological changes in podocytes directly. Therefore, zebrafish are a promising tool to investigate gene-drug interactions. Our results demonstrated that zebrafish are a suitable model to identify new drug targets on podocytes in future studies. This species are more convenient and costs less than rodents.

**Figure 8:**
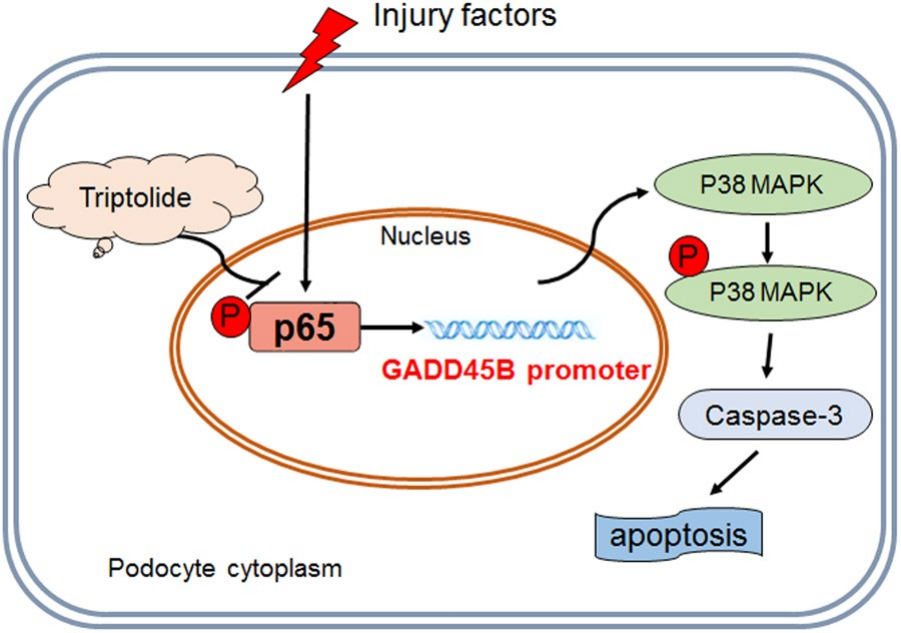
Schematic diagram of triptolide downregulation of *GADD45B* expression in the protection of podocytes from apoptosis. Podocyte injury produces remarkable *GADD45B* upregulation due to aberrant phospho-NF-κB p65 induction and *GADD45B* promoter transactivation. *GADD45B* induces podocyte apoptosis via activation of the p38 MAPK signalling pathway. Triptolide effectively corrects aberrant NF-κB activation and decreases *GADD45*B expression to protect podocytes from apoptosis.

In conclusion, our results provide new insights into the potential molecular mechanisms of triptolide protection of podocytes. We demonstrated that triptolide protected podocytes from apoptosis and that this effect was associated with inhibition of the NF-κB/*GADD45B*/p38 MAPK signalling pathway (Fig. 8). We also demonstrated that zebrafish embryos are a robust experimental vertebrate model that can be used to identify new podocyte-protecting drugs and perform fast and reproducible pharmacological assays with excellent outcomes.

## Materials and Methods

### Reagents

Triptolide (C20H24O6, molecular weight 360, Nanjing Spring & Autumn Biological Engineering Company, Nanjing, China). Metronidazole (MTZ) and PAN (Sigma-Aldrich, St Louis, MO, USA). The anti-zebrafish podocin antibody was synthesized by Genscript Company (Nanjing, China), and the sequence of the synthesized peptides was CDGSDDGTKDSPM.

### Fish Breeding and Maintenance

Zebrafish (Danio rerio) were maintained under standard laboratory conditions^28^. All experiments on zebrafish were performed in accordance with the guidelines for the Care and Use of Laboratory Animals approved by the Nanjing University School of Medicine.

### Generation of transgenic zebrafish

Transgenic zebrafish lines Tg(*pod:Gal4*), Tg(*UAS:NTR-mCherr*y), Tg(*l-fabp:VDBP*-GFP), Tg(*UAS:gadd45ba*,*cmlc*_*2*_*:gfp*) and Tg(*UAS:gadd45bb*,*cml*_*c2*_*:gfp*) were generated as previously described^4^.

### Drug administration

The Tg(*pod:gal4:UAS:NTR-mCherry*) embryos at 60 hpf were selected according to mCherry fluorescence in glomeruli under a stereomicroscope (Nikon SMZ25, Tokyo, Japan) and transferred into 6-well plates. Embryos were divided randomly into normal control, triptolide control, and MTZ model and MTZ + triptolide treatment group. Thirty embryos were pooled together in each well. The triptolide treatment group and control group were pretreated with triptolide (20 ng ml^−1^) for 24 h prior to MTZ treatment and throughout the experimental process. The normal control and MTZ model groups were incubated in embryo medium containing 0.1% DMSO. MTZ was added to the MTZ model and MTZ + triptolide treatment groups to induce podocyte injury at 84 hpf for the indicated period.

### Proteinuria Assay

The *l-fabp:VDBP*-GFP plasmid was a kind gift from Dr. Weibin Zhou. Triple transgenic embryos Tg(*pod:Gal4;UAS:NTR-mCherry;lfabp:VDBP-GFP*) were generated by crossing *Tg*(*pod:Gal4;UAS:NTR-mCherry)* and *Tg(lfabp: VDBP-GFP)* fish. Three embryos were pooled in each well of a 96-well plate in 150 µl of E3 medium for each group. MTZ treatment was initiated at 120 hpf. The E3 medium in each group was collected for proteinuria assays using ELISA (Abcam, Cambridge, UK) at 144 hpf^10^.

### Whole-mount Immunofluorescence and Quantitation

Immunohistochemistry was performed as described previously^29^. The anti-zebrafish podocin antibody (1:10000) and anti-cleaved caspase 3 antibody (cat 559565, 1: 1000; BD Pharmingen, CA, USA) were incubated for 10–15 h at 4 °C. The secondary antibody was donkey anti-rabbit IgG (H + L) conjugated with Alexa Fluor® 488 (cat A-21206, 1:1000, Invitrogen, USA). Confocal images were captured using a Zeiss LSM 710 laser-scanning microscope and Z-stack. We scanned 5–10 glomeruli images in each group using consistent parameters and performed a maximum intensity projection with confocal microscope to quantify fluorescence intensities^30^. The average value of the glomeruli images from each group was used as the protein expression state. The mean immunofluorescence intensity, which measured as the average grey level (GL), and the area ratio of the stained area to the glomerulus area (AR)^31^ were examined using Image Pro Plus 6.0.

### Transmission Electron Microscopy (TEM)

The TEM was performed according to our previous study^32^.

### Cell culture and reagent treatment

The conditionally immortalized human podocytes (HPCs) were a gift from Dr. Moin Saleem at Bristol University, UK. The cells were cultured in RPMI 1640 medium containing streptomycin, penicillin, insulin, transferrin, selenite (Gibco, USA) and 10% foetal calf serum at 33 °C. Podocytes were switched to a growth temperature to 37 °C for 10–14 days to induce a differentiated phenotype. Podocyte injury was induced with 50 μg ml^−1^ PAN for the indicated time. Triptolide (10 ng ml^−1^) was preincubated for 30 min prior to PAN exposure.

### Annexin V flow cytometric analysis of apoptosis

Podocytes were collected, washed with ice-cold PBS twice, resuspended in 100 μl of 1 × binding buffer, and then incubated with 5 μl Annexin V-FITC and 5 μl PI staining solution (Vazyme, Nanjing, China) at room temperature for 10 min, followed by analysis with FACScan using Cellquest software (Becton Dickinson, Franklin Lakes, NJ, USA).

### Microarray analysis

Total RNA was extracted using TRIzol (Invitrogen) and purified using an RNeasy mini kit (QIAGEN). A NanoDrop spectrophotometer (ND-1000, NanoDrop Technologies) was used to evaluate RNA quality and quantity. RNA integrity was examined using gel electrophoresis. Labelling, hybridization, washing and staining were performed according to manufacturer’s recommendations (Affymetrix, Santa Clara, CA, USA). The microarray analysis was performed using the Affymetrix Human HTA2.0.

### RNA interference

NF-κB p65 knockdown was performed using an NF-κB p65-specific siRNA. The sequence of the sense strand of the siRNA oligonucleotides was 5-GCCCUAUCCCUUUACGUCA-3′. Podocytes differentiated well and were cultured to ~90% confluence prior to transfection with NF-κB p65 siRNA using Lipofectamine RNAiMAX (Invitrogen, USA) following the manufacturer’s instructions.

### Western blot

Lysates from cultured cells were prepared and subjected to Western blot assays following previously described procedures^4^. Western blot was performed using the following primary antibodies: anti-*GADD45B* rabbit polyclonal antibody (Abcam, Cambridge, UK), NF-κB p65, phospho-NF-κB p65 (Ser536) and phospho-IκBα (Beyotime Bio Co., Shanghai, China). Western blots were developed using an ECL plus Western blotting detection system (Vazyme, USA). The protein quantities were analysed using Image J software.

### Chromatin immunoprecipitation (ChIP)

The chromatin immunoprecipitation (ChIP) assay was performed using the Zymo Research ChIP Chromatin Immuno-precipitation Kit (CA, USA) following the manufacturer’s instructions. Antibodies (5 μg) specific for phospho-NF-κB p65 or an IgG isoform control were incubated at 4 °C overnight with rotation. PCR amplification was performed after de-crosslinking and extracting the DNA using primer sets that contained the tentative NF-κB p65 binding elements in the *GADD45B* promoter region (ChIP-pos F/R, +255 to +455). The primer sequences are provided in Supplement Table 1.

### Luciferase reporter assay

The 39-UTR of *GADD45B* was obtained from PCR using human genomic DNA and inserted downstream of the pGL3-promoter (Promega, Madison, WI). Reporter plasmids were transfected into cultured human podocytes. A renilla luciferase plasmid (pRL-SV40, Promega, USA) was used as an internal control. The NF-κB p65 siRNA and con-siRNA were co-transfected with luciferase plasmids using Lipofectamine 2000 (Invitrogen, USA). A dual luciferase assay system (Promega, USA) was used to assess luciferase activity after 24 h.

### Statistical analysis

Statistical analyses were performed using SPSS software (version 20). All results are expressed as the means ± SD. We used the unpaired t-test to compare two groups and one-way ANOVA to compare three or more groups. Statistical significance was accepted at P < 0.05.

### Data Availability

The datasets generated during and/or analysed during the current study are available from the corresponding author on reasonable request.

### Approval

All sampling and experimental procedures involving animals were performed according to the experimental animal use guidelines and approved by the Institutional Animal Care and Use Committee at Jinling Hospital (Nanjing, Jiangsu, China). Approved number: 2014GJJ-110.

## Electronic supplementary material

Supplementary Material
