# Supplementary material for: Triptolide attenuates proteinuria and podocyte apoptosis via inhibition of NF-κB/GADD45B
Source: Sci Rep. 2018 Jul 18;8:10843. doi: 10.1038/s41598-018-29203-1 (PMC6052061; doi:10.1038/s41598-018-29203-1)
Supplement: Supplementary file 1 — Supplementary Material [file 41598_2018_29203_MOESM1_ESM.doc]

**Triptolide attenuates proteinuria and podocyte apoptosis via inhibition of NF-κB /*GADD45B***

**Ling Wang^1^, Liwen Zhang^1^, Qing Hou^1^, Xiaodong Zhu^1^, Zhaohong Chen^1^*, Zhihong Liu^1^***

^1^National Clinical Research Center of Kidney Diseases, Jinling Hospital, Nanjing University School of Medicine, Nanjing, 210016, China

Correspondence: Zhaohong Chen, [rin@nju.edu.cn](mailto:rin@nju.edu.cn); Zhihong Liu, liuzhihong@nju.edu.cn


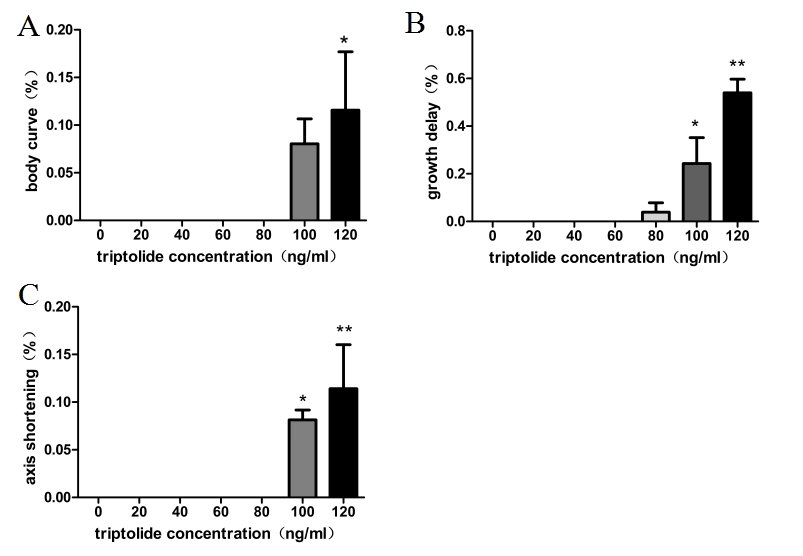


Supplemental Figure1. Rates of development abnormalities in zebrafish embryos treated with triptolide at different concentration (0, 20, 40, 60, 80, 100 and 120ng ml^-1^). Embryos treated with triptolide at concentration more than 80 ng ml^-1^ displayed evident body curve (A), growth delay (B) and axis shortening (C). Data are expressed as mean±SD. **P<0.01;*P<0.05. (On the basis of three triplicate tests).

Supplemental table 1.The primer sequences.

| gadd45ba qF  gadd45ba qR | 5’-ACTGCATCCTCGTCACTAACTC-3’  5’-TTTTGCAACGGCTCTCCTCA-3’ |
| --- | --- |
| gadd45bb qF  gadd45bb qR | 5’-TGTTACTAACCCCCAAGCCG-3’  5’-GGCAATAGAAGGCACCCAC-3’ |
| Zebrafish GAPDH qF  Zebrafish GAPDH qR | 5’-CCAAGGCTGTAGGCAAAGTA-3’  5’-GACTGTCAGATCCACAACAGAG-3’ |
| GADD45B qF  GADD45B qR | 5’-AACATGACGCTGGAAGAGCT-3’  5’-AGAAGGACTGGATGAGCGTG-3’ |
| Human GAPDH qF  Human GAPDH qR | 5’-GGGAAACTGTGGCGTGAT-3’  5’-GTGGTCGTTGAGGGCAAT-3’ |
| GADD45B Chip-pcr qF  GADD45B Chip-pcr qR | 5’-GCCAAGTTGATGAATGTGTGAG-3’  5’-GCAGACGATACATCAGGATACG-3’ |
| GADD45B promoter F-sacI  GADD45B promoter R-hindIII | 5’-ctatcgataggtaccgagctcAAGGCTGGAACGCCAGGC-3’  5’-cagtaccggaatgccaagcttCTGAGGGGGCCAAAGGGT-3’ |


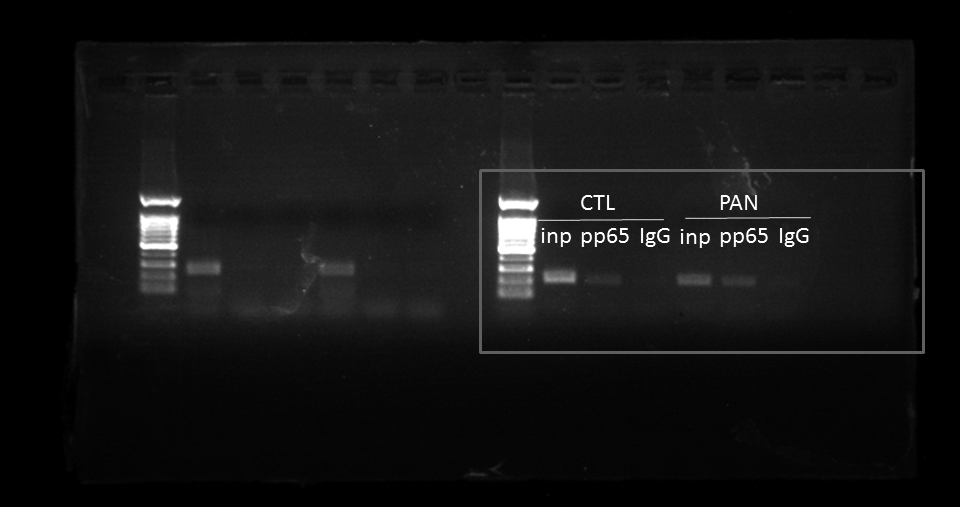


**Supplemental Figure2. Full length gel for Figure 7.**
